# Antifungal and antibiofilm activities of bee venom loaded on chitosan nanoparticles: a novel approach for combating fungal human pathogens

**DOI:** 10.1007/s11274-022-03425-y

**Published:** 2022-10-25

**Authors:** Samia E. El-Didamony, Mohamed H. Kalaba, Esmail M. El-Fakharany, Mahmoud H. Sultan, Mohamed H. Sharaf

**Affiliations:** 1 Zoology and Entomology Department, Faculty of Science, Al-Azhar University (Girls), Nasr City, Cairo, 11884 Egypt; 2 Botany and Microbiology Department, Faculty of Science, Al-Azhar University (Boys), Cairo, 11884 Egypt; 3 Protein Research Department, Genetic Engineering and Biotechnology Research Institute, City of Scientific Research and Technological Applications, New Borg El Arab, 21934 Alexandria Egypt

**Keywords:** Bee venom-loaded chitosan nanoparticles, *Cryptococcus neoformans*, *Kodamaea ohmeri*, *Candida albicans*, Biofilm formation, Yeast-hyphae transition

## Abstract

The prevalence of opportunistic human fungal pathogens is increasing worldwide, and antimicrobial resistance is one of the greatest medical challenges the world faces. Therefore, this study aimed to develop a novel agent to control fungal pathogens. The honeybee products (honey, royal jelly, propolis, bee bread, and bee venom) were screened against unicellular fungal (UCF) pathogens (*Cryptococcus neoformans*, *Kodamaea ohmeri*, and *Candida albicans*) and the bee venom was only exhibited an inhibitory effect against them. The protein contents of crude bee venom were separated using the gel filtration technique into eight fractions which were visualized on sodium dodecyl sulfate–polyacrylamide gel electrophoresis (SDS–PAGE) to confirm the presence of five bands with molecular weights of 65, 43, 21, 15, and 3 KDa. Bee venom (BV) of *Apis mellifera* loaded chitosan nanoparticles were prepared by the ionotropic gelation method. The encapsulation efficiency%, average size, zeta potentials, and surface appearance by Transmission electron microscope (TEM) were evaluated for the prepared nanoparticles. The minimum inhibitory concentration (MIC) of crude BV and BV loaded chitosan nanoparticles (BV-CNPs) was evaluated against the offer mentioned UCF where the MIC values of crude BV were 6.25, 3.12 & 6.25 while MIC values in the case of BV-CNPs were decreased to 3.12, 3.12 & 1.56 mg/ml against *C. neoformans*, *K. ohmeri* and *C. albicans*, respectively. Also, the results showed that BV-CNPs suppressed the biofilm formation as well as yeast to hyphal transition formed by the examined UCF. These results revealed that BV-CNPs are a promising natural compound for fungal pathogens treatment.

**Graphical abstract:**

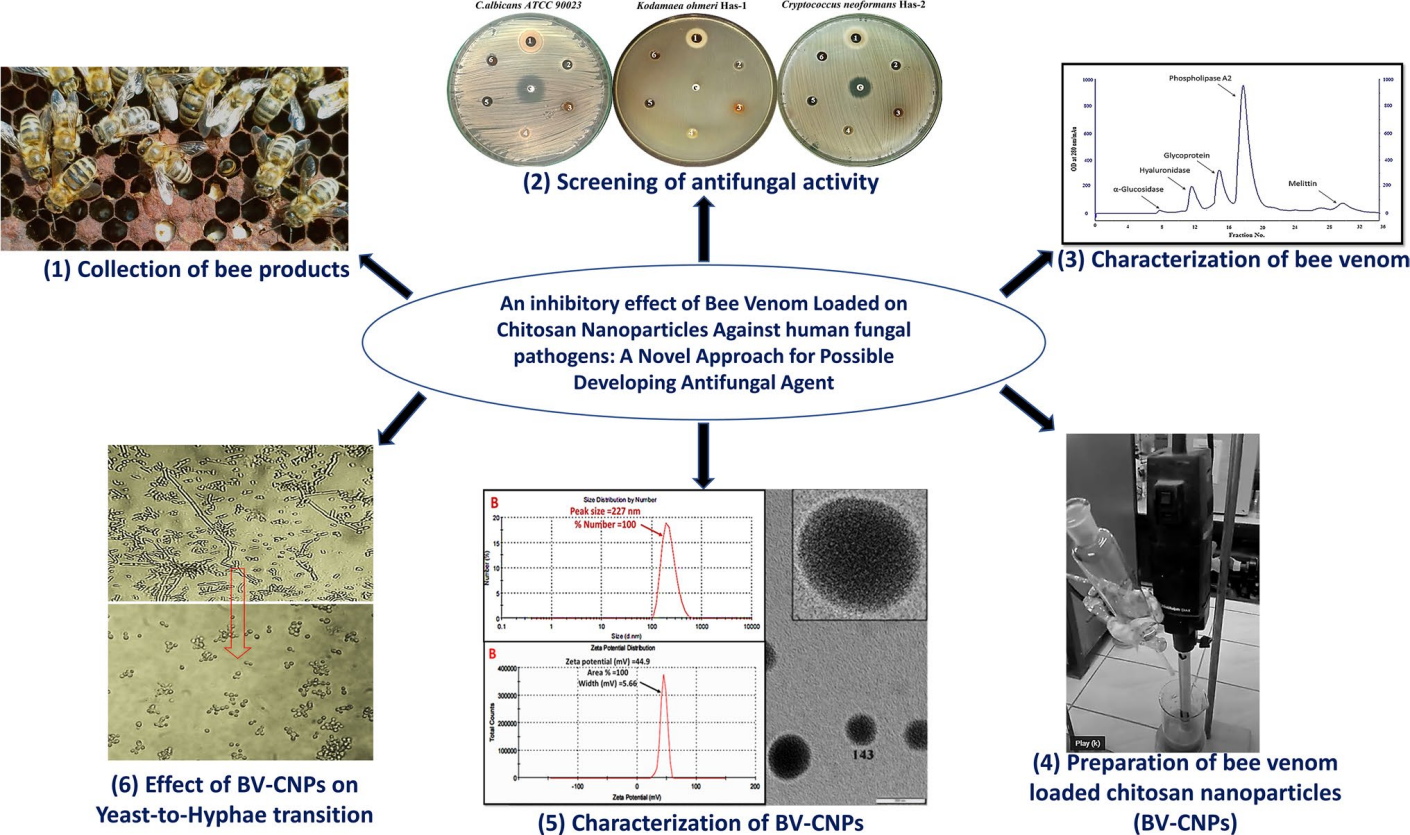

## Introduction

Pathogenic fungi have a huge influence on human health. Fungal infections represent one of the most prevalent forms of human infection (Fisher et al. 2020). Certain fungal species can cause invasive diseases in humans, these infections have been estimated to kill 1.5 million and affect a billion people per year (Bongomin et al. 2017; Firacative 2020). These life-threatening infections are caused primarily by species of the *Candida*, *Cryptococcus*, and *Pneumocystis* genera, which cause life-threatening infections in individuals with impaired immunity or other underlying conditions. These infections can be broadly divided into environmentally (e.g., *Cryptococcus* and *Kodamaea*) or endogenously (e.g., *Candida*) acquired (Malavia et al. 2017).

*Candida albicans* is the most prevalent and prominent clinical fungal pathogen. It causes significant mortality in populations, mainly in immunocompromised, neutropenic patients, which stay for long time in intensive care units (Cheng et al. 2012). This fungus is frequently found in healthy people’s oral cavities and gastrointestinal tracts, but it can cause serious disease (Talapko et al. 2021). According to epidemiological studies, *Candida* species are the fourth cause of bloodstream infections in hospitals, with a death rate of up to 71% (Bac et al. 2019; Raja 2021). Other fungi, such as non-albicans *Candida* species and *Cryptococcus* sp., have emerged as vital infection agents (Schieffelin et al. 2014). All cases of cryptococcosis in people and animals are caused by *Cryptococcus neoformans*. Cryptococcosis is a disease that results in a lung infection that may spread to the brain, causing meningoencephalitis. In 2009, *C. neoformans* was estimated to cause more than 1 million cases of meningitis primarily in HIV-infected patients of whom more than 600,000 deaths. Unfortunately, In 2016 this number of cases increased to about 16.7 million people (Hagan 2018). In 1998, *Kodamaea ohmeri* was isolated for the first time from a patient’s blood (Bergman et al. 1998). Afterward, it emerged as a human pathogen capable of causing life-threatening infections, particularly in immunocompromised people. Human infections caused by this organism have been recorded sporadically over the world, including fungemia, endocarditis, catheter-related bloodstream infection, and cutaneous infection, among several others (Ni et al. 2018; Yu et al. 2020). Moreover, it has been reported that significant mortality of up to 50% is caused by invasive infections of *K. ohmeri* (Zhou et al. 2021).

With the increasing occurrence of fungal infectious diseases, the treatment of them remains a major challenge for human health, due to the difficulty in achieving a sustainable solution and the high likelihood of recurrence. Whereas systemic antifungal therapy is limited to azoles (itraconazole and fluconazole), terbinafine, and other antifungal agents (LaSenna and Tosti 2015), and griseofulvin (Kreijkamp et al. 2017). Also, the major challenges in treating fungal infections include increasing multidrug resistance and harmful consequences. Furthermore, the recent rise of opportunistic fungus infections has emphasized the need for new antifungal agents (Terra et al. 2014).

Therefore, we need to get new compounds with a novel mode of action to get through the current antifungal resistance mechanism in fungi. Natural compounds of both plant and animal origin have conventionally been used in the medicinal field because of their wide range of therapeutic action, including antibacterial, antifungal, and antiviral activity (Newman and Cragg 2020). Among these natural compounds are bee products such as honey, bee pollen, royal jelly, propolis, beeswax, beebread, and bee venom, which serve as good sources for a new drug with therapeutically potential in the management of various cancer types and infections by different types of bacteria, viruses, and parasites (Cornara et al. 2017).

*Apis mellifera* venom is composed of a complex mixture of active peptides, enzymes, and amines (Hider 1988). Bee venom (BV) has been used to treat diverse disorders (Hegazi 2012). Modern pharmacological studies showed that in vitro and in vivo, BV has antibacterial effects against bacteria, viruses, and fungi (El-Seedi et al. 2020). However, the use of B.V. has shown adverse effects on normal cells as reported by Gülmez et al. (2017), Cherniack and Govorushko (2018). Thus, there is still a need for more studies that can eliminate or even decrease the cytotoxic effect of B.V. and enhance its therapeutic effect in the target organ. Nanotechnology has been involved in most biomedical fields, including nanomaterial applications themselves as antimicrobial, anticancer, antiviral, and biocidal agents, or their loading with bioactive drugs/compounds to increase their solubility, stability, functionality, and delivery to the human body (Sezer 2014; Beyth et al. 2015). Among these nanoparticles, the natural biopolymer chitosan possessed nontoxic, biocompatible, and biodegradable properties. Also, chitosan possessed the ability to safely deliver of BV for their evaluation as anticancer agents (Moselhy et al. 2017; Alalawy et al. 2020).

In an attempt to improve pathogenic fungal therapeutic protocols, this study was carried out to screen honeybee products (honey, royal jelly, propolis, bee bread, and bee venom) against *C. neoformans*, *K. ohmeri*, and *C. albicans*. Also, the study developed and evaluated the use of cross-linked chitosan nanoparticles as a controlled drug carrier system for effective and safe delivery of the most effective product, bee venom.

## Materials and methods

### Collection of bee products

Honeybee products (Bee venom, propolis, royal jelly, honey and bee bread) collection were carried out at the apiary of Apiculture Department, Plant Protection Research Institute, Agriculture Research Center, during the 2019 spring and summer seasons.

#### Bee venom

Honeybee venom was collected from healthy workers of *Apis mellifera* (L.) according to Fakhim-Zadeh (1998), El-Didamony et al. (2022) by the electric shock device (VC-6 F model from Apitronic Services, 9611 No. 4 Road, Richmond, B.C., Canada). Two beehives were used, each one containing approximately ten thousands of bee workers. The fresh bee venom was packed in dark glass tubes and stored at a temperature of − 4 °C until use.

#### Propolis

The propolis sample was collected by using transparent glass slides, these slides were arranged contiguous to each other and were put onto the top bar of the combs, with an elevation at approximately 3 mm in between as described by Aly (2012) and obtained propolis was kept desiccated in the dark until processing. After that, the crude propolis sample was soaked in 80% ethanol and stirred overnight for 5days using magnetic stirrer (FALC F600 in Treviglio, Italy) in dark conditions. Then, the resulting product was filtered through Whatman (No.1) filter paper to obtain clear supernatant. A rotary evaporator under reduced pressure of 450 mm Hg at 40 °C was used to concentrate the clear supernatant and the residue was stored in the dark glass bottle at room temperature until use (Cunha et al. 2004).

#### Royal jelly

Royal jelly was harvested from the honeybee colony according to Wu et al. (2015). Royal jelly was collected without grafting larvae. The collection method is based on the insertion of a special plastic comb foundation matched with supporting larva devices and plastic cell cups into a colony to allow a queen to lay eggs in this comb. The frames of plastic cell cups with larvae were placed within the productive colony to extract royal jelly. The collected royal jelly was stored in vials and was kept in the refrigerator till use.

#### Honey

A honey sample was collected from a honeycomb in framed beehive according to Sammataro and Avitabile (1998). Briefly, all the capping of capped cells in which honey was stored were removed manually using a heated knife. The frames were placed in a honey extractor through which the honey is removed by centrifugal force, then the resulting honey was passed through a screen to collect clean liquid honey.

#### Bee bread

Bee bread was collected manually from the beehives. The frame of bees was separated from the hive and the bees were removed from the frame with a brush. Then, the fame was carried to the lab for the extraction of the bee bread. Bee bread was gathered with the help of a spatula and collected upon butter paper. The bee bread was stored in Eppendorf and was kept in the refrigerator till use. This method was reported by Kumar (2019).

### Isolation of unicellular fungal (UCF) isolates

According to the differences in the morphological characteristics of their cultures, two UCF isolates of clinical origin were selected and obtained from Elmkhtabar lab at Eldoky, Giza, Egypt. These isolates were coded as Has-1 and Has-2 and subjected to identification using an automated Vitek2 system (Vitek2-YST ID card, bioMerieux SA, France) as described in the manufacturer’s instructions.

### Screening of antifungal activity of bee products

Honeybee products were screened for antifungal activity according to Kumar et al. (2015) with minor modification against the obtained unicellular fungal isolates Has-1 and Has-2 as well as a reference strain of *C. albicans*—ATCC 90028 using the agar well diffusion method. The tested UCF were maintained on Sabouraud Dextrose Agar (Merck, Germany) plates at 4 °C and sub-cultured before each experiment. These UCF were suspended in sterile Sabouraud Dextrose broth (SDB, Merck, Germany) and turbidity was adjusted to 0.5 McFarland standard. The agar well diffusion assay was performed as follows: SDA plates were flooded with 100 µl of SDB media containing culture suspensions (1 × 10^6^ CFU/ml) of the tested UCF and the suspensions were evenly spread. The plates were dried for 20 min followed by punching a hole (8 mm) with a sterile corn borer in the plates. The stock solutions of each one of the honeybee products were freshly prepared by dissolving it using distilled water at a concentration of 20 mg/ml and sterilized using a 0.22 micro m syringe filter (Sigma-Aldrich, Darmstadt, Germany) before use and placed in the wells. Twenty-five microliters of the sterilized bee products were placed in each well in addition to Fluconazole paper discs (25 µg) that were included as an antifungal control. Plates were placed in the refrigerator for 2 h. before incubation at 35 °C for 24 h. The growth of UCF was observed and diameters of zones of inhibition were recorded after incubation time. The experiment was repeated three times and the mean values were calculated.

### Characterization of bee venom

To separate and fractionate the components of the crude bee venom, a gel filtration chromatography technique was used. The crude sample (150 mg) was dissolved in 3 ml of 50 mm phosphate buffer pH 7.2, which was then centrifuged at 10,000 rpm for 20 min to remove the impurities. The filtered sample of bee venom was applied to a Sephacryl S100 column (16/60, 120 ml, GE Health care, Sweden) previously equilibrated with the same buffer. Fractions were eluted with 50 mm phosphate buffer pH 7.2 containing 0.15 M NaCl using FPLC (AKTA Prime plus, GE Healthcare, Sweden) at a flow rate of 0.5 ml/min and fraction size of 4 ml/fraction. The optical density of eluted fractions was measured at 280 nm and the molecular weight of the crude sample and eluted fractions were estimated by SDS-PAGE.

### Sodium dodecyl sulfate–polyacrylamide gel electrophoresis (SDS–PAGE)

To determine the protein patterns of bee venom based on their molecular weights, the separating gel (13.5%) and stacking gel (5%) were prepared according to the procedure of (Laemmli 1970). Ten microliters of the eluted fractions as well as the crude bee venom were loaded onto the gel in comparison with 5 µl of protein standard (Protein molecular mass markers) for the determination of the molecular weight of each protein.

### Preparation of chitosan nanoparticles and bee venom cross-linked chitosan nanoparticles

The ionotropic gelation method was used to prepare chitosan nanoparticles by mixing chitosan cations with sodium tripolyphosphate (TPP) anions according to the procedure described by (Taher et al. 2017). Chitosan (1 mg/ml) was dissolved in 1.5% acetic acid aqueous solution. Sodium tripolyphosphate was dissolved in deionized water at a concentration of 1 mg/ml. The TPP solution was then added to the chitosan solution in drops. Rapid homogenization (~ 20,000 rpm) in an ice bath for 60 min resulted in the spontaneous formation of nanoparticles. The bee venom-loaded nanoparticles were formed by the addition of venom at a concentration of 20 mg/ml to the TPP solution before the incorporation of the chitosan solution. Finally, nanoparticles were separated by centrifuge at 11,000 rpm and 4 °C for 90 min; lyophilized then stored at 4 °C.

### Characterization of nanoparticles

#### Determination of encapsulation efficiency

The amount of venom encapsulated in the nanoparticles was measured by calculating the difference between the total amounts of the venom added in the nanoparticle preparation and the amount of non-entrapped venom remaining in the supernatant. The samples were centrifuged at 11,000 rpm and 4 °C for 90 min and the amount of bee venom was evaluated as a total protein according to Bradford (1976) at 595 nm. After that the venom encapsulation efficiency (AE) of nanoparticles was calculated as follows:

$$\% {\text{AE}} = \left[ {{{\left( {{\text{A}} - {\text{B}}} \right)} \mathord{\left/ {\vphantom {{\left( {{\text{A}} - {\text{B}}} \right)} {\text{A}}}} \right. \kern-\nulldelimiterspace} {\text{A}}}} \right] \times {\text{1}}00,$$

where A is the total amount of venom, B is the free amount of venom in the supernatant.

#### Determination of the particle size and zeta potential

The particle size and size distribution of the freshly prepared chitosan nanoparticles (empty) and bee venom-chitosan NPs (BV-CNPs) were evaluated by Zetasizer (Malvern Instruments, UK), based on the dynamic light scattering (DLS) technique in Dynamic Light Scattering Lab., Nanotechnology Center, Egyptian Petroleum Research Institute. Particle size was reported as a number mean diameter (NMD). Also, the zeta potential was measured by the same instrument.

#### Transmission electron microscopy

The morphology of empty chitosan nanoparticles and (BV-CNPs) was investigated by transmission electron microscope (TEM) at the Faculty of Science, Al-Azhar University (Boys branch), Cairo, Egypt. The samples were fixed on copper grids and stained with 1% phosphotungstic acid solution (PTA). The specimens were air-dried at room temperature and examined using Philips 400 transmission electron microscope (Netherlands) at an accelerating voltage of 80 kV.

### Determination of minimum inhibitory concentration (MIC) of bee venom-chitosan NPs

The MIC of bee venom-chitosan NPs (BV-CNPs) and crude bee venom (CBV) was performed according to El-Sherbiny et al. (2017). In brief, cell suspensions of UCF isolates and *C. albicans* ATCC 90023, were prepared at 1 × 10^6^ CFU/ml in RPMI 1640 medium (Sigma) supplemented with 0.2% (w/v) glucose. One hundred microliters aliquots of these cell suspensions were dispensed into 96-well microtiter plates. The tested materials were tested in a twofold serial dilution. The BV-CNPs and CBV were added to RPMI1640medium into wells at a final concentration ranging from 25 to 0.390 mg/ml. Wells containing negative control (medium + BV-CNPs or CBV at the tested concentrations) were performed to determine the differences in optical density (OD). The plate was incubated for 24 h at 37 °C, and the absorbance was measured at 620 nm using a microplate reader (Biorad mod 680). MIC was defined as the lowest concentration of the BV-CNPs or CBV able to inhibit the visible growth of microorganisms.

### Evaluation of the antibiofilm activity of BV-CNPs

The anti-biofilm activity of the BV-CNPs was determined using a 96-well microtiter plate (sterile, flat-bottom polystyrene, Merck, Germany) at concentrations ranging from MIC to 1/8 MIC against UCF isolates and *C. albicans* ATCC 90023 according to Barapatre et al. (2016) with some modification. Individual wells of the plates were filled with 100 µl of RPMI1640 medium containing cell suspensions of 1 × 10^6^ cells/mL and subsequently, 100 µl of BV-CNPs of the corresponding concentrations to each microorganism were added and thoroughly mixed. Then, the prepared test plates were incubated in a static condition at 37℃ for 24 h. Mixtures without tested microorganisms were used as a negative control. After that, we discarded the contents of the well plates and gently washed them with phosphate-buffered saline (PBS, pH 7.2) to eliminate free-floating non-adherent cells from the wells. The microtiter plates’ wells were then air-dried for 45 min. After drying, adherent “sessile” cells of UCF isolates in the wells were fixed with absolute alcohol, and the wells were then flooded with crystal violet stain (0.1%, w/v) and incubated in the dark for 30 min. Afterward, the wells were thoroughly washed with sterile deionized water until all excess dye was removed. The plates were then air-dried again. After the well plates dried completely, 200 ml of 33% acetic acid was added to each well. Finally, the absorbance was measured at 620 nm and the inhibition of biofilm formation percentage was calculated using the following equation:

$${\text{Biofilm}}\;{\text{inhibition}}\;\% = 1 - \left( {\frac{{{\text{OD620}}\;{\text{of}}\;{\text{cells}}\;{\text{treated}}\;{\text{with}}\;{\text{BV-CNPs}}}}{{{\text{OD620}}\;{\text{of}}\;{\text{the}}\;{\text{control}}}}~} \right) \times 100$$

### Effect of BV-CNPs on yeast-to-hyphae transition morphology

Spider medium (1% nutrient broth, 1% mannitol, 0.2% K_2_HPO_4_, pH 7.2) was used to explore the effects of BV-CNPs on the yeast-to-hyphal morphological transition according to Sharaf (2020). The cell suspension of UCF was adjusted at a concentration of 1 × 10^6^ CFU/ml in spider medium and transferred 200 µl into each well of 96-well plates. Then UCF were treated with different concentrations (one quarter, one half, and entire MIC value) of BV-CNPs corresponding to each strain in addition to control (the treatment was replaced with distilled water). After incubation at 37 °C for 8 h, the morphologies of cells exposed to different concentrations of BV-CNPs were examined under Zeiss inverted microscope (Germany).

### Statistical analysis

The examinations were performed in three replicates and the data were represented as mean ± standard error mean (SEM) using Sigmaplot 12.5 and Microsoft office 365.

## Results

### Identification of unicellular fungal isolates and antifungal activity of bee products

Using the vitek2 automated system, both Has-1 and Has-2 unicellular fungal isolates were identified as *K. ohmeri* and *C. neoformans*, with 97 and 95% probability and confidence levels of excellent and very good, respectively. In addition to these isolates, *C. albicans* ATCC 90023 was used in this study as a standard strain for the evaluation of the antifungal activity of the bee products.

**Fig. 1:**
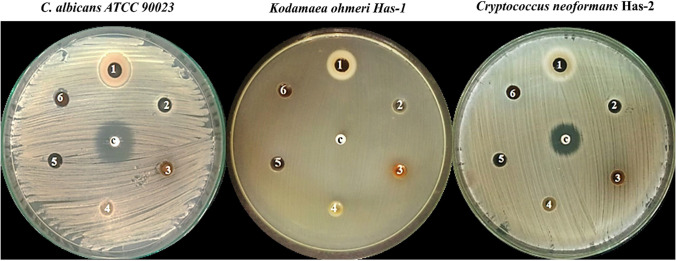
Antifungal activity of bee products against *C. albicans* ATCC 90023, *K. ohmeri*, and *C. neoformans*. 1: bee venom, 2: honey, 3: propolis, 4: bee bread, 5: Royal jelly, 6: DMSO and C: fluconazole

The effects of various bee products on the previously mentioned species were investigated, and it was found that bee venom is the sole effective product against them, with inhibition zone diameters ranging from 18 to 26 mm (Fig. 1). Furthermore, fluconazole (an antifungal control) had no inhibitory impact on *K. ohmeri*. It also affected the growth of *C. neoformans* and *C. albicans* ATCC 90023 with an inhibition zone diameter of 20 and 28 respectively (Table 1).

**Table 1:** Antifungal activity of bee products against unicellular fungal clinical isolates using agar well diffusion assay

Treatments	Mean ± standard error values of the inhibition zone diameter (mm)		
	*C. albicans* ATCC 90023	*C. neoformans*	*K.a ohmeri*
Bee venom	26.3 ± 1.2	23 ± 0.66	18 ± 0.88
Propolis	0	0	0
Royal jelly	0	0	0
Honey	0	0	0
bee bread	0	0	0
Control solvent (DMSO)	0	0	0
Fluconazole	28 ± 0.66	20 ± 1.15	0

### Characterization of bee venom

#### SDS–PAGE of bee venom protein patterns

**Fig. 2:**
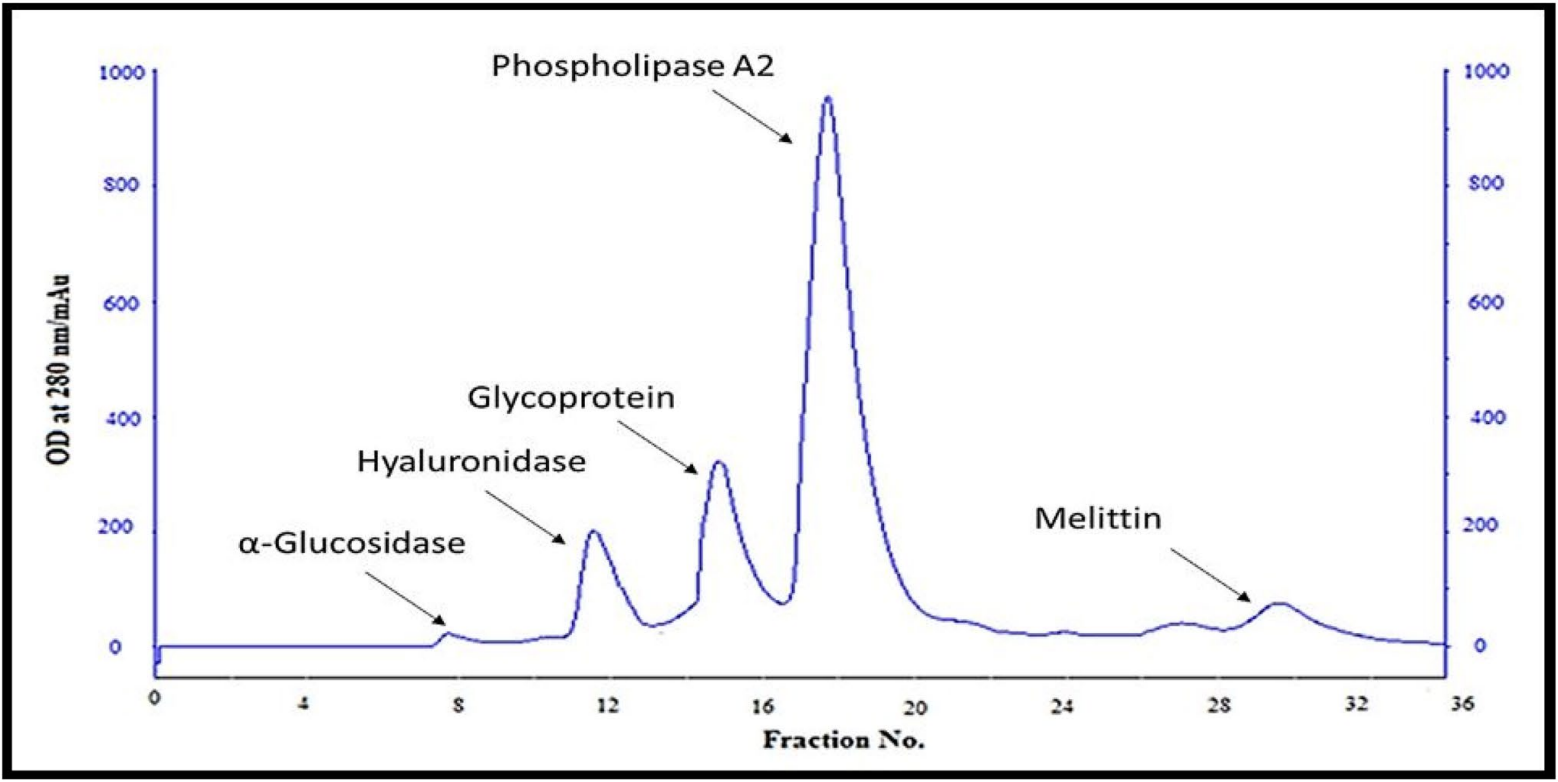
A typical elution profile for the chromatography of crude bee venom on Sephacryl 100 column previously equilibrated with phosphate buffer pH 7.2. The elution was performed using phosphate buffer pH 7.2 containing 0.15 M NaCl at flow rate 0.5 ml/fraction and absorbance recorded at 280

**Fig. 3:**
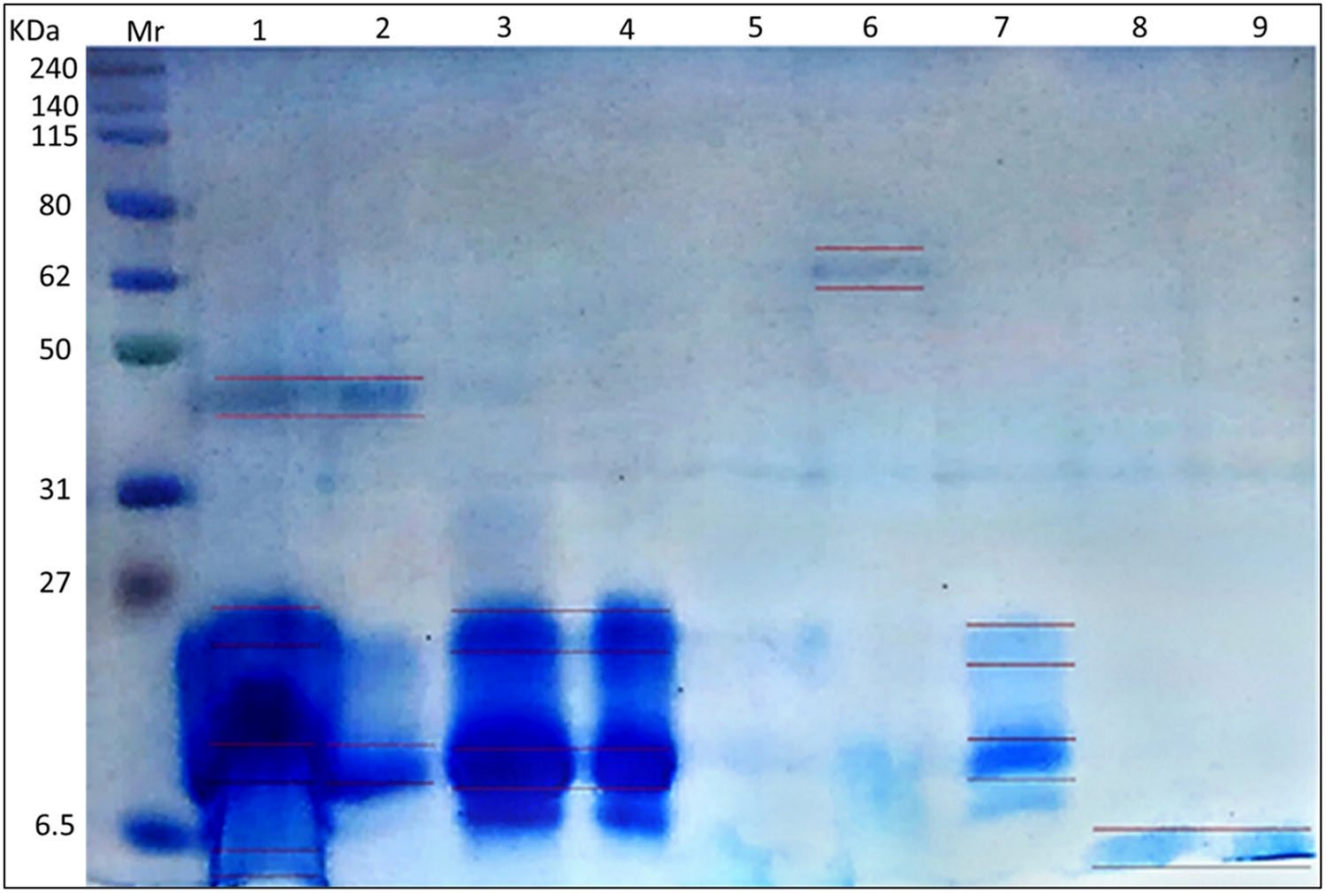
13.5% SDS–PAGE of bee venom after fractionation by Sephacryl 100 column. Lane Mr is molecular mass markers, lane 1 is crude bee venom, and lanes 2–9 are the eluted fractions respectively. The total protein profile of fractions was analyzed by SDS–PAGE and stained with Coomassie Brilliant Blue

Figure 2 represents the typical bee venom elution profile following fractionation with a Syphacryl S100 column (size exclusion chromatography). According to the findings, bee venom had been separated into five components based on their molecular weight, as indicated by five peaks on the chromatogram. These peaks were represented by eight fractions: 7–9, 11–12, 13–14, 15–16, 17–18, 19–20, 20–21, and 28–30 in addition to crude bee venom on SDS–PAGE. In evaluating peptide bands revealed by electrophoretic analysis (Fig. 3), we discovered that the eluted fractions contained five bands with molecular weights of 65, 43, 21, 15, and 3 KDa. These bands were also present in the crude BV sample except for the band with a molecular weight of 65, which was not present while it was presented only in infraction no. 6. On the other hand, the bands of 15 and 21 KDa were repeated in fractions 3, 4, and 7 with different intensities.

### Characterization of nanoparticles

In the present study, the BV-CNPs were prepared at (1 mg/ml chitosan, 1 mg/ml TPP and 20 mg/ml of venom), and their encapsulation efficiency was 96.55% (Table 2).

**Table 2:** Characterization of free chitosan nanoparticles and BV-CNPs

Samples	Entrapment efficiency %	Particle size (nm)	Polydispersity index	Zeta potential (mV)
A	–	99.56	0.270	50.3
B	96.55	227.20	0.374	44.9

*A* free chitosan nanoparticles, *B* BV-CNPs

**Fig. 4:**
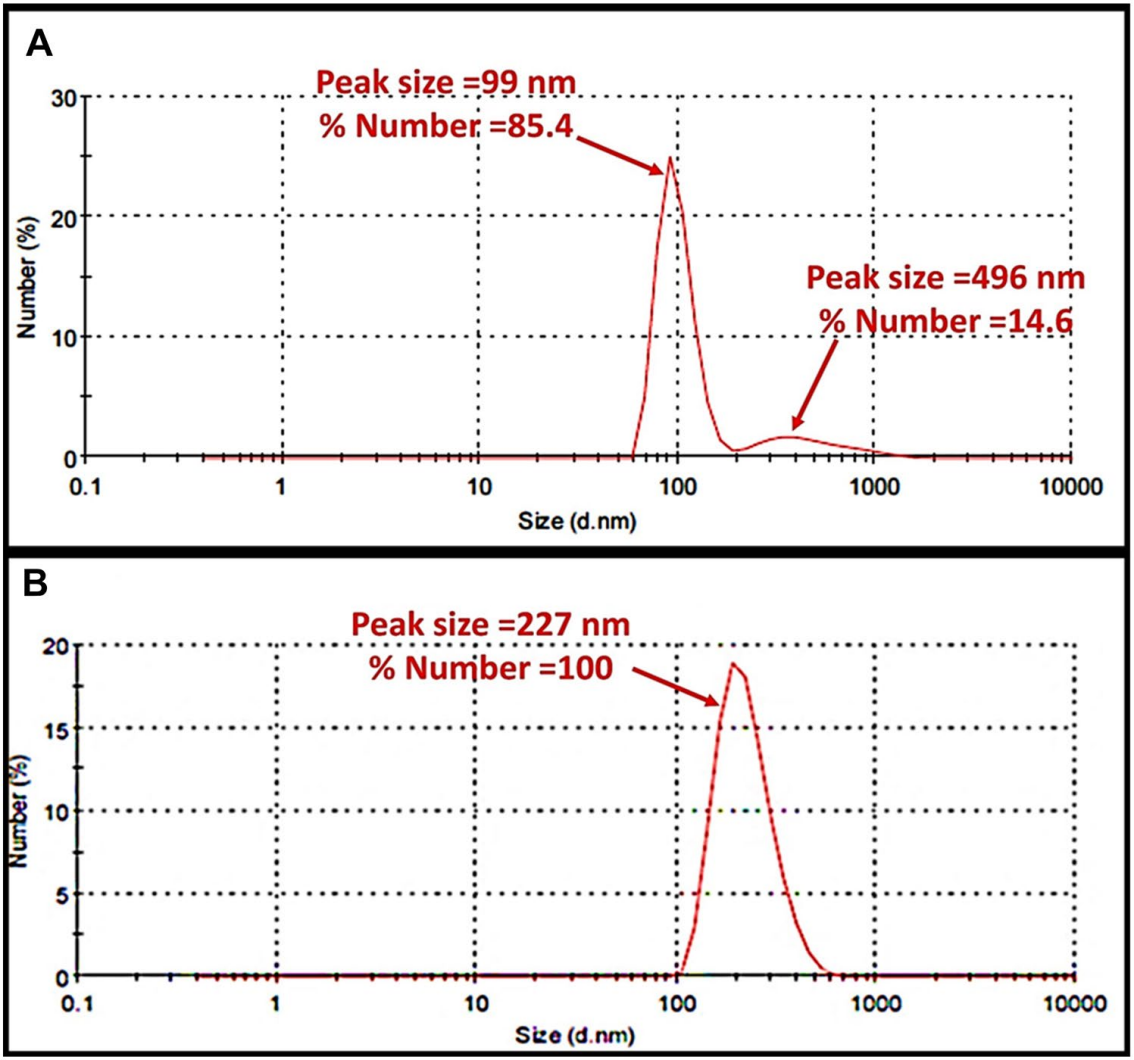
Size distribution by % number of **A f**ree chitosan nanoparticles and **B** bee venom-loaded chitosan nanoparticles (BV-CNPs)

The average size of free chitosan nanoparticles and BV-CNPs were estimated in their colloidal using the dynamic light scattering technique. The results obtained by Zetasizer revealed that the venom-loaded nanoparticles are larger than the chitosan nanoparticles without bee venom in their size where the average size of free chitosan nanoparticles and BV-CNPs was 99.56 and 227.2 nm, respectively. The polydispersity index (PDI) value of chitosan nanoparticles was 0.270 while that of BV-CNPs was 0.374, indicating a narrow and favorable particle size distribution (PDI < 0.5) (Fig. 4A, B).

**Fig. 5:**
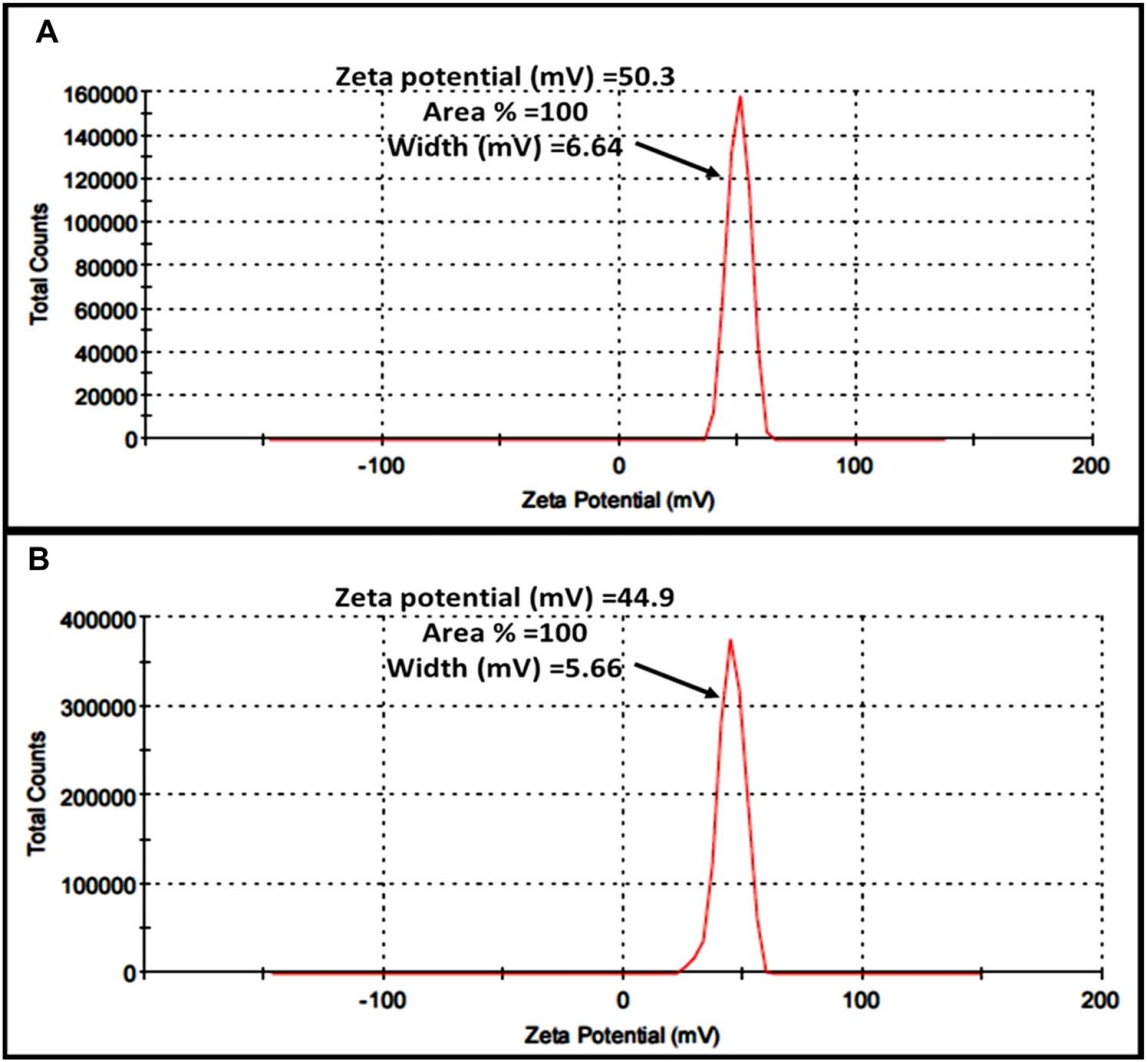
Zeta potential of **A** free chitosan nanoparticles and **B** bee venom-loaded chitosan nanoparticles (BV-CNPs)

The obtained results illustrated that the values of zeta potentials were 50.3 and 44.9 mV of chitosan nanoparticles and venom-loaded chitosan nanoparticles, respectively (Fig. 5A, B).

**Fig. 6:**
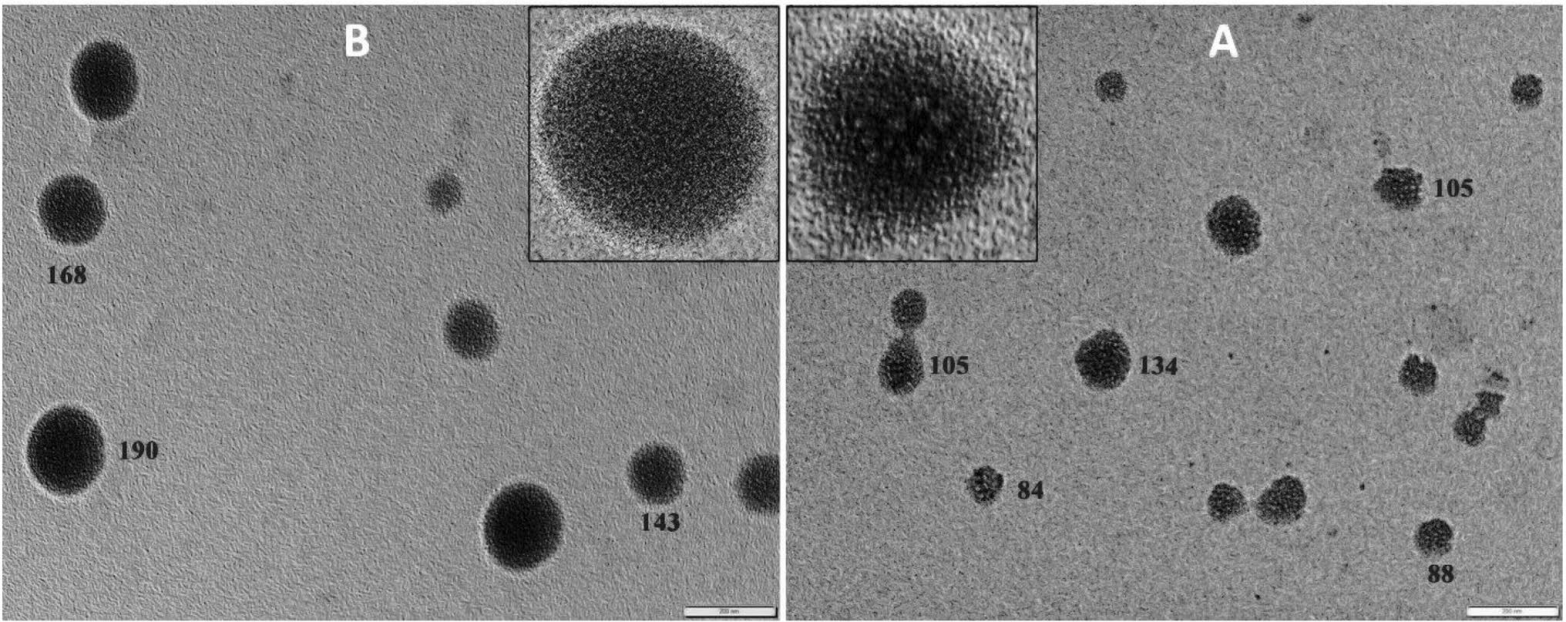
Transmission electron micrographs of **A** free chitosan nanoparticles and **B** BV-CNPs

Transmission electron microscope (TEM) micrographs have shown the morphological properties and surface appearance of nanoparticles. Both the free chitosan nanoparticles and the BV-CNPs seemed dispersed spherical and free of aggregations. There are some differences seen in the micrographs including particle size, which increased in BV-CNPs, and particle surface, which seemed softer in BV-CNPs and surrounded by a light layer that was missing in free chitosan nanoparticles (Fig. 6A, B).

### Determination of MIC value of BV-CNPs

A standardized broth microdilution technique was used to determine the MIC of BV-CNPs and CBV. The results showed that the MIC value of BV-CNPs against the tested microorganisms was lower than that of CBV, except for *K. ohmeri*, which had the same MIC value of 3.125 mg/ml for both BV-CNPs and CBV, as shown in (Table 3). According to this data, we used BV-CNPs only for further studies against the same microorganisms.

**Table 3:** MIC values of free chitosan nanoparticles (CBV) and BV-CNPs

Microorganism	MIC (mg/ml)	
	CBV	BV-CNPs
*C. neoformans*	6.25	3.125
*K. ohmeri*	3.125	3.125
*C. albicans* ATCC 90023	6.25	1.562

### Anti-biofilm activity of BV-CNPs

**Fig. 7:**
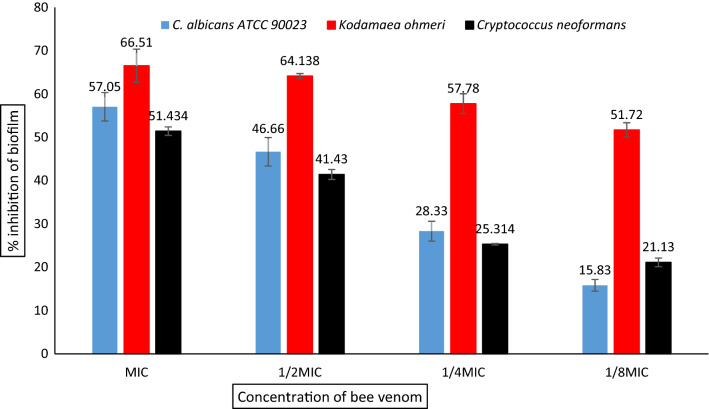
Antibiofilm activity of BV-CNPs

The results showed that the effect of BV-CNPs on biofilm formation varied amongst the three studied microorganisms, although it was all concentration-dependent. Although *K. ohmeri* had the maximum percentage of suppression of biofilm formation at MIC concentration (66.51%), with a drop in 1/8 MIC concentration, the inhibitory impact of biofilm formation remained at 51.72%. In the case of *C. albicans* ATCC 90,023 and *C. neoformans*, the percentages of suppressing biofilm formation with the highest MIC were 57.05 and 51.43%, and this percentage was reduced to 15.83 and 21.13%, respectively while using 1/8MIC (Fig. 7).

### Inhibition of yeast-hyphal transition

**Fig. 8:**
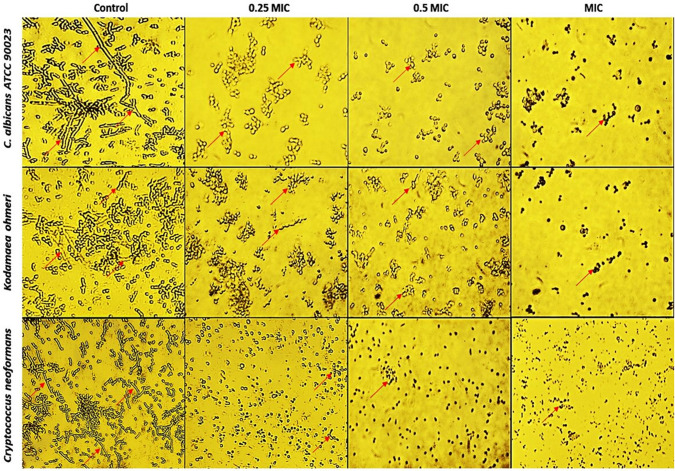
Inhibition of hyphal transition in spider media using different concentrations of BV-CNPs under the inverted microscope (Magnification power = ×100)

The results showed that all three microorganisms examined were able to make a yeast-to-hypha transition in the same medium (spider media) but to varying degrees. For example, the capacity of *C. albicans* ATCC 90023 to make a yeast-to-hypha transition looked to be higher than that of *K. ohmeri*, while *C. neoformans* appeared to be lower than the two. The yeast-to-hypha transition capacity of these microorganisms was lost when they were treated with a quarter of the MIC value corresponding to each of them, but many cells remained in the form of clusters or attached cells in randomized forms, which are called pseudo-hyphae. The presence of this pseudo-hyphae was dramatically decreased when cells were treated at a dose of 0.5 MIC in both *C. albicans* ATCC 90023 and *K. ohmeri*, and less so in *C. neoformans*. All kinds of yeast-to-hypha transition, as well as pseudo-hyphae, disappeared in all examined microorganisms when cells were treated at a greater dose, which is the entire MIC value. The cells seemed tiny and atrophied to a great extent when compared to lower doses or even the control, possibly indicating death (Fig. 8).

## Discussion

For the effective treatment of dermatophyte infections, new antibacterial or antifungal agents with fewer side effects must be developed (AL-Khikani and Ayit 2021). The antibacterial activities of bee venom have been also intensively explored as a natural antibacterial agent however, the use of bee products in the treatment of infectious diseases caused by *Candida* is extremely restricted (Lee 2016). In the current study, among the tested bee products, bee venom only exhibited a considerable inhibitory effect against the tested strains. These results may be attributed to the following scenario: inside the fungal cell, ROS production is controlled by antioxidant production. When this equilibrium is disrupted (for example, by the presence of bee venom), ROS buildup can cause oxidative damage to lipids, proteins, and DNA, ultimately leading to cell death. On the other side, fluconazole was used in our study as positive control and did not show an inhibitory effect against *K. ohmeri* which is considered resistant to it according to Clinical and laboratory Standards Institute (CLSI) (2008). These results agreed with (Yu et al. 2012) who reported that dermatophytosis, which occurs via *Trichophyton mentagrophytes* and *T. rubrum* fungi can be inhibited by bee venom treatment, while fluconazole (commercial antifungal drugs) did not prevent the development of the same pathogens. The study and our findings may prove that bee venom was more potent than fluconazole.

In our study, the band with molecular weights of 65 KDa was high molecular weight and might be α-Glucosidase (Hossen et al. 2016), while the band of 43 KDa may be indicated to be hyaluronidase. According to (Zidan et al. 2018), the molecular weight of hyaluronidase ranges from 35 to 53 KDa depending on the carbohydrate quantity bound to the protein moiety. The band with molecular weights ~ 20 KDa was detected in different eluted fractions. Mammadova and ShA (2017) purified and identified the components of bee venom using ion-exchange chromatography with a concentration gradient from 0.01 to 1 M sodium chloride solution on a Servacel-52 column and detected bands, including one with a molecular weight of 22 that they classified as glycoprotein. The band with molecular weight ranging from 19 to 14 KDa might be forms of phospholipase A2 (PLA2) (Pucca et al. 2019; Darwish et al. 2021), and the band with a molecular weight of ~ 3 kDa which was detected by this technique may confirm the presence of melittin in our venom specimens. This result was matched with previous studies which reported that the bulk of insect venoms that sting humans are composed of various chemical combinations. This complex mixture consists of amino acids (aa), peptides, proteins, enzymes, carbohydrates, biogenic amines, volatile compounds, phospholipids, and pheromones (Suh et al. 2015; Sangboonruang et al. 2020; Carpena et al. 2020).

The present results indicate that the prepared BV-CNPs recorded a high encapsulation efficiency of 96.55%. This high value may be due to the dissolution of venom in TTP solution at the moment of cross-linked nanoparticle formation, these protein molecules are trapped inside the polymeric matrix of chitosan nanoparticles (Gan and Wang 2007). Moreover, the electrostatic interactions between positively charged groups of chitosan and negatively charged proteins are frequent during the formation of nanoparticles and other parts adsorbed on the surface of nanoparticles (Gan et al. 2005). These results matched with previous reports, for instance, Soares et al. (2012) recorded a high value of encapsulation efficiency that is greater than 90% for *Tityus serrulatus* scorpion venom loaded on chitosan nanoparticles. Also, Taher et al. (2017) found that bee venom-loaded chitosan nanoparticles which were prepared at (1 mg/ml CS and1mg/ml TPP with 300 µg/ml of venom) and recorded an encapsulation efficiency of 96.26%.

The obtained results showed that the loading of bee venom led to increasing the size of chitosan nanoparticles. Increasing the size of chitosan nanoparticles containing bee venom in comparison to those that do not contain it may demonstrate the potential of nano-chitosan particles to carry bee venom (Rocha Soares et al. 2012). Mostly, nanocomposites are manufactured for successful biomedical applications in a size range from 20 up to 300 nm, concerning the minimum particles that could spread to any part of the body (Krug and Wick 2011). This finding corresponds well with (Taher et al. 2017) who reported that the size of venom-loaded nanoparticles prepared at a concentration of chitosan 1 mg/ml have a particle size of 140.3 nm while venom-loaded nanoparticles have a size of 187 nm. Alalawy et al. (2020) showed that the chitosan nanoparticles had a smaller particle size range from 92.1 to 157.3 nm than BV chitosan nanocomposites which had a particle size range from 147.3 to 269.6 nm.

Our results demonstrated that the venom loading led to a reduction of the particle’s zeta potential. This reduction can be due to electrostatic interaction between the carboxyl groups on the surface of the venom molecule and amine groups at certain sites of the chitosan molecule. But venom molecule attachment did not sufficiently suppress all the positive surface charge of chitosan molecules, Therefore, It still seems that a high proportion of free amine groups on the chitosan chain remained unoccupied. Similar results were obtained by Mohammadpour et al. (2012b) who demonstrated that the zeta potential of chitosan nanoparticles only and *Mesobuthus eupeus* scorpion venom-loaded nanoparticles were 50.2 mV and 44.1 mV, respectively. Also, Taher et al. (2017) found that zeta potential values of chitosan nanoparticles and BV-loaded nanoparticles were 43.7 mV and 36.1 mV, respectively.

The current results demonstrated that TEM micrographs for both the free chitosan nanoparticles and the BV-CNPs were spherical but particle size was increased in BV-CNPs, and surrounded by a light layer. The layer may be responsible for the increase in the hydrodynamic size of BV-CNPs than the free chitosan nanoparticles. According to this result, TEM may approve the interactions between chitosan nanoparticles and BV through these differences in the morphological characteristics of nanoparticle types. The results were in agreement with Mohammadpour et al. (2012a) who observed that the shape of the nanoparticles was approximate to spheres with an almost homogeneous structure when *Hemiscorpius lepturus* Scorpion venom loaded on chitosan nanoparticles. Also, Taher et al. (2017) found that the chitosan nanoparticles had a spherical shape, and it was observed a layer around the nanoparticles’ core bee venom-loaded nanoparticles.

In general, the results show that the entrapment process influences the efficacy of bee venom since the MIC values for BV-CNPs were lower than for CBV, which might be related to the following hypothesis: The CBV concentrations used in the experiment represent a single dosage to which the tested microorganisms were exposed at the start of the experiment only, which may have resulted to the tolerance of some of these concentrations by the end of the period, resulting in relatively high MIC values. On the other hand, the BV-CNPs entrapment kept it available sustainably in moderate amounts throughout the experiment, which may suppress the ability of the tested microorganisms to tolerate it, and this may be lower BV-CNPs MIC values. This hypothesis may be supported also by previous reports such as Sahoo et al. (2007), who stated that nanotechnology interference in most biological applications has increased, including nanomaterial applications such as antimicrobial, anticancer, antiviral, and biocidal agents, or their loading with bioactive drugs/compounds to increase their solubility, stability, functionality, and delivery to their targets. Nano polymers, notably Nano-chitosan, were among the most researched nanomaterials for their bioactivity and utility as drug carriers, antibacterial, anticancer, and gene delivery agents, either alone or in combination with other active substances (Gan and Wang 2007; El Rabey et al. 2019). This led us to perform a preliminary investigation to examine the effect of CNPs, BV-CNPs, and CBV. This revealed that there was no effect by empty nano-chitosan particles, thus it was not included in the MIC measurement.

Microorganisms may form biofilms in nature in addition to planktonic growth, and these biofilms enable microbial cells to proliferate in the host environment and spread to colonize new areas. Fungal biofilms are made up of adhering cells that are surrounded by an extracellular matrix. *C. albicans*, the most frequent fungal infection in hospitals, has well-documented biofilm development. Furthermore, biofilms of *C. neoformans* are among the leading causes of nosocomial infections caused by the fungus (Oshiro et al. 2019). In addition, *K. ohmeri* is one of the species isolated from the oral cavity of immunocompromised individuals and shown to generate biofilm on abiotic surfaces (Ferreira et al. 2019). In the current study, BV-CNPs were evaluated for biofilm inhibition against UCF isolates and demonstrated a high effect against all tested species, including *C. albicans* ATCC 90023, *C. neoformans*, and *K. ohmeri*. Even though the mechanism of biofilm formation in unicellular fungi varies depending on the species (Kim and Kim 2021), inhibition of biofilm formation in UCF isolates and *C. albicans* ATCC 90023 by BV-CNPs suggest that these species may share a common mechanism to induce the biofilm, but more research is needed.

Unicellular fungi (Yeasts) are generally individual budding oval-shaped cells with several microns in diameter. On the other hand, hyphae are elongated cells that are joined end to end. Hyphal filaments are generally around 2 μm in diameter, with parallel-sided walls and no septal constrictions. Pseudo-hyphal filaments, on the contrary, are somewhat larger in diameter (2.8 μm), lack parallel sides, and contain constrictions at septal junctions and the mother-bud neck (Kadosh 2017). The capacity of yeasts, particularly *C. albicans* and *C. neoformans* to make a reversible morphological shift from yeast to filamentous form is often associated with pathogenicity and is critical for a range of virulence-related activities (Lee et al. 2013; Hu et al. 2021). Thus, the ability of BV-CNPs to inhibit the formation of filamentous forms was evaluated and the results revealed that the examined UCF was able to form both true and pseudo-hyphae as well as single forms at different conditions. When a quarter or half MIC value was employed, BV-CNPs inhibited the development of both true and pseudo-hyphae, in addition to the appearance of tiny-sized single cells and potentially atrophy when the MIC value was utilized. These findings may be implied that BV-CNPs not only affect hyphal development in *C. albicans* ATCC 90023, *K. ohmeri*, and *C. neoformans*, but also interfere with yeast form proliferation. The morphological transformation from yeast to hyphae cells has previously been shown to be critical in several processes, including biofilm formation (Tobaldini-Valerio et al. 2016). Thus, the ability of BV-CNPs to suppress yeast-hyphae transition may be related to their ability to prevent biofilm development.

## Conclusion

We can be concluded that bee venom has activity against clinical fungal isolates *K. ohmeri*, and *C. neoformans* as well as a reference strain of *C. albicans*—ATCC 90028. The results described herein provide significant enhancement of bee venom activity against clinical fungal isolates and a reference strain when it is loaded on chitosan nanoparticles under the conditions implemented in the current study. Besides, the antibiofilm activity tested in this work exhibit anti-virulence activity against human pathogen fungi. Our findings in this research, BV-CNPs offer promising treatments for infectious illnesses with unicellular fungi.

## Data Availability

Not applicable.
